# Nonlinear association between perceived social support and fear of disease progression in stroke patients: a cross-sectional study

**DOI:** 10.3389/fpubh.2026.1954246

**Published:** 2026-10-13

**Authors:** Yijia Qi, Yuyan Yang, Xiaohui Liu, Xiaoping Yang, Shailing Ma, Yingjie Zheng, Jing Li, Jiajia Lai

**Affiliations:** 1 School of Nursing, Ningxia Medical University, Yinchuan, Ningxia, China; 2 General Hospital of Ningxia Medical University, Yinchuan, Ningxia, China

**Keywords:** stroke, perceived social support, fear of disease progression, nonlinear association, restricted cubic splines

## Abstract

**Objectives:**

To explore the nonlinear association between perceived social support and fear of disease progression in stroke patients.

**Methods:**

In this cross-sectional study, 300 patients with stroke completed a general information questionnaire, the Fear of Progression Questionnaire-Short Form (FoP-Q-SF), the Multidimensional Scale of Perceived Social Support (MSPSS), and the Modified Barthel Index (MBI). Independent-samples *t* tests, one-way analysis of variance, hierarchical linear regression, and restricted cubic spline (RCS) analysis were used.

**Results:**

The mean FoP score was 30.34 ± 8.56. In the hierarchical regression model, higher PSS was associated with lower FoP (*B* = −0.223, *P* < 0.001). Compared with employed participants, unemployed participants had lower FoP scores (*B* = −3.188, *P* = 0.008), and severe ADL dependence was associated with higher FoP compared with independence (*B* = 4.921, *P* = 0.035). The restricted cubic spline analysis indicated evidence of nonlinearity (*P*_foroverall_ < 0.001; *P*_for_ _nonlinear_ = 0.023). Exploratory piecewise linear regression identified an estimated change point at a PSS score of 67 (*P* = 0.011). Below 67, each one-point increase in PSS was associated with a 0.319-point lower FoP score (*B* = −0.319, 95% CI −0.396 to −0.241; *P* < 0.001). At PSS scores ≥ 67, the slope was not statistically significant and was imprecisely estimated (*B* = 0.304, 95% CI −0.142 to 0.751; *P* = 0.182).

**Conclusions:**

This study suggested a nonlinear association between perceived social support and fear of disease progression, with a change point at a perceived social support score of 67. However, this change point requires validation in larger multicenter and prospective studies. In addition, unemployed and severely dependent were independently associated with fear of disease progression. These findings may help guide more comprehensive psychosocial assessment for stroke patients at risk of fear of disease progression.

## 1 Introduction

Stroke, also referred to as a cerebrovascular accident, is a group of acute cerebrovascular disorders characterized by the “four highs”: high incidence, high disability rate, high mortality rate, and high recurrence rate (1). In 2021, there were approximately 11.9 million new stroke cases and 93.8 million prevalent stroke cases worldwide. Stroke is the second leading cause of death and the third leading cause of disability-adjusted life years (DALYs) among non-communicable diseases globally (2). The World Stroke Organization projects that the number of stroke-related deaths globally will increase by 50% between 2020 and 2050, rising from 6.6 million to 9.7 million annually (3). The burden of stroke is also substantial in China. According to the Global Burden of Disease (GBD) study, there were approximately 4.09 million new stroke cases in China in 2021, and the number of prevalent stroke cases ranked highest in the world (4). It is also the leading cause of death and disability among adults in China (5). Studies have shown that 70%−80% of stroke survivors experience residual disabilities after treatment (6). Moreover, many studies have reported that the recurrence rate of stroke ranges from approximately 10% within one year (7) to 12% within five years (8). The persistent risk of recurrence and long-term sequelae are also associated with substantial psychological burden, including anxiety, depression, and personality changes (9, 10). More importantly, there is chronic distress driven by fear–namely, fear of disease recurrence, disability progression, and disease progression (FoP) (11, 12). These psychological difficulties may hinder patients from achieving successful recovery. Therefore, the risks of recurrence and persistent disability may contribute to FoP after stroke (13).

Fear of disease progression (FoP) is an individual's apprehension about the biological, psychological, and social implications of disease progression or recurrence when confronted with an existing disease (14). Mehnert et al. originally developed the Fear of Progression-Questionnaire-Short Form (FoP-Q-SF) in 2006 (15), and Wu translated and validated it into Chinese in 2015 (16). Studies have shown that nearly half of stroke survivors experience dependence on others (17) and difficulty in returning to previous life roles and activities after stroke (18). The items of the FoP-Q-SF-such as the fear of dependency on others and worry about being unable to resume daily activities–are conceptually relevant to the core concerns of stroke survivors, and the scale has since been used in stroke populations (19, 20). Previous research on FoP has primarily focused on cancer patients. In recent years, however, FoP has increasingly been recognized as a clinically relevant psychological concern in stroke populations. A cross-sectional study by Liu et al. (19) reported that approximately 45.6% of stroke patients experience FoP. More importantly, studies have found that FoP is associated with poorer quality of life and lower adherence to treatment and rehabilitation (21, 22).

Perceived social support (PSS) refers to an individual's subjective appraisal of the availability and adequacy of resources provided by their social networks (23). Studies have shown that good PSS can help patients face and cope with difficulties with a positive and optimistic attitude, reduce psychological stress, and promote positive psychological adjustment (24, 25). In addition, social support and social-network functioning are associated with depression, health-related quality of life, participation, and recovery-related outcomes after stroke (26, 27). Consistent with this broader literature, recent stroke studies have reported inverse cross-sectional associations between social support and FoP (28, 29). These findings support PSS as a plausible psychosocial correlate of FoP. However, the vast majority of previous studies examining the association between FoP and PSS in stroke patients have employed traditional linear regression or related linear modeling techniques (28, 29), assuming a simple linear relationship between the two variables. In real-world clinical settings, however, the association between PSS and FoP may not be adequately represented by a simple linear model (30). A nonlinear association would allow the magnitude and direction of the association to vary across the PSS distribution. Restricted cubic spline (RCS) is a flexible nonlinear analytical tool that characterizes the shape of the association between a continuous variable and an outcome by placing multiple knots at different quantiles of the data distribution and fitting piecewise polynomial functions. Compared with conventional methods, RCS preserves the full information of the variable, allows a more accurate description of the curvature of the dose-response relationship, and uses nonlinearity tests to determine whether the linear assumption holds (31). Although RCS has been applied in nonlinear dose-response studies in some areas (32), evidence regarding its use in examining the association between PSS and FoP in stroke patients remains lacking. Therefore, this study aimed to explore the nonlinear association between PSS and FoP in stroke patients using RCS and threshold effect analyses, and to identify an exploratory change point after adjusting for confounders, to provide exploratory evidence for future research and the development of intervention strategies.

## 2 Materials and methods

### 2.1 Design

This was a descriptive cross-sectional study, reported in accordance with the STROBE statement for cross-sectional research (33).

### 2.2 Participants

This study employed a convenience sampling method to recruit stroke patients who were admitted to the Department of Neurology at the General Hospital of Ningxia Medical University and the First People's Hospital of Yinchuan, Ningxia Hui Autonomous Region, China, between May and September 2023. Convenience sampling was used because recruitment was restricted to eligible inpatients at the two participating hospitals during the predefined study period. The two participating hospitals are tertiary teaching hospitals located in the same region.

The inclusion criteria were: Patients diagnosed with stroke who met the diagnostic criteria proposed in the Chinese Guidelines for the Diagnosis and Treatment of Intracerebral Hemorrhage 2019 (34) and the Chinese Guidelines for the Diagnosis and Treatment of Acute Ischemic Stroke 2018 (35), and confirmed by CT or MRI; Age ≥ 18 years; Clinically stable, conscious, and able to complete the questionnaire independently or with assistance from the researchers.

The exclusion criteria were: Patients with severe systemic comorbidities, such as heart failure or malignant disease; Those who had previously suffered from or were currently diagnosed with psychiatric and psychological disorders; Patients unable to complete the assessment because of severe aphasia and hemiplegia.

According to the rule of thumb that the sample size should be 10–20 times the number of independent variables, a total of 12 independent variables were included in this study. After accounting for a 20% rate of invalid questionnaires, the required sample size was estimated to range from 150 to 300 participants. Because RCS regression was additionally used to explore potential nonlinear associations, the adequacy of the sample size was also considered in relation to model complexity. Candidate models with 3 to 5 knots were considered. The present study ultimately selected 4 knots, corresponding to 3 degrees of freedom, with 1 representing the linear component and 2 representing the nonlinear component. With 300 participants included in the analysis, the sample size was considered reasonable for exploratory modeling of the nonlinear association.

### 2.3 Survey instruments

#### 2.3.1 General information questionnaire

The general information questionnaire consisted of demographic and disease-related information. Demographic variables included patients'gender, age, place of residence, educational level, marital status, work status, household economic status, and method of medical payment. Disease-related variables included stroke type and level of knowledge about stroke, assessed using a single self-reported question: “Are you familiar with stroke-related information?” with four response levels: very familiar, moderately familiar, slightly familiar, or unfamiliar.

#### 2.3.2 Fear of progression-questionnaire-short form (FoP-Q-SF)

The fear of progression-questionnaire-short form (FoP-Q-SF), developed by Mehnert et al. (15), translated into Chinese by Wu et al. (16), was used to assess participants' FoP. The scale has been used in stroke populations (19, 20). It comprises two subscales: physical health and social-family. The scale includes 12 items rated on a 5-point Likert scale, with total scores ranging from 12 to 60, with higher scores indicating higher levels of FoP. The Cronbach's α coefficient of the FoP-Q-SF in this study was 0.848.

#### 2.3.3 Multidimensional scale of perceived social support (MSPSS)

The multidimensional scale of perceived social support (MSPSS), developed by Zimet et al. (36), translated into Chinese by Jiang (37), was used to measure PSS. The scale comprises 12 items across three subscales: family support, friend support, and other supports. Responses are rated on a 7-point Likert scale, with total scores ranging from 12 to 84. Higher scores indicate greater social support. The Cronbach's α coefficient of the MSPSS in this study was 0.927.

#### 2.3.4 Modified Barthel Index (MBI)

Activities of daily living (ADL) are daily home-based activities that people carry out to maintain health and wellbeing (38). In this study, ADL was assessed using the Chinese version of the Modified Barthel Index (MBI) (39), which has demonstrated validity and reliability in stroke populations (40). It includes 10 items covering feeding, bathing, grooming, dressing, bowel and bladder control, toilet use, transfers, walking, and stair climbing. The total score ranges from 0 to 100, with higher scores reflecting greater functional independence. Scores of 100, 61–99, 41–60, and 0–40 were classified as independent, mildly dependent, moderately dependent, and severely dependent, respectively.

### 2.4 Data collection and quality control

All eligible patients admitted to the participating departments during the study period were consecutively invited to participate. Trained researchers conducted the survey face-to-face. Before distributing the questionnaire, the researchers explained the study purpose and procedures to the patients and obtained written informed consent. Data collection followed standardized instructions. Patients completed the questionnaires independently, if a patient had difficulty, the researcher could assist by reading the questions one by one. All questionnaires were collected and checked on site. Any incomplete or erroneous questionnaires were supplemented or corrected immediately. During the recruitment period, 308 eligible patients were approached, and all agreed to participate. A total of 308 questionnaires were returned. Of these, 8 questionnaires were excluded because they showed obvious response patterns, and 300 valid questionnaires were retained. There were no missing values in the analytic dataset, yielding a valid-questionnaire rate of 97.4%.

### 2.5 Statistical analysis

Analyses were performed using SPSS version 27.0 and R version 4.6.0 (rms package). Continuous variables were presented as mean ± standard deviation (M ± SD), while categorical variables were described as frequencies and percentages. In univariate analyses, independent-samples *t* tests or one-way ANOVA were used to compare continuous variables between groups, in order to identify potential factors associated with FoP. Variables with *P* < 0.05 in univariate analyses were entered into the multivariate linear regression model. Model 1 was adjusted for the variables selected based on the above criteria. Model 2 additionally included PSS as the primary explanatory variable. In addition, a sensitivity analysis was performed to assess the robustness of the findings. Regression assumptions were assessed before interpretation of the models, including residual normality using Q-Q plots and homoscedasticity using residual plots. Additionally, to further examine the nonlinear relationship between PSS and FoP, RCS analysis was performed within the linear regression framework, adjusting for covariates that were statistically significant in the multivariate regression models. Candidate models with 3 to 5 knots were compared using the Akaike information criterion (AIC), and the model with 4 knots, placed at the 5th, 35th, 65th, and 95th percentiles of the PSS distribution, was selected as the final model based on the minimum AIC. Nonlinearity was assessed by testing the nonlinear spline terms. An exploratory two-piecewise linear regression model was then fitted to estimate a change point in the association between PSS and FoP, and the fit of the one-line and two-piecewise models was compared using a likelihood-ratio test. All tests were two-sided, with *P* < 0.05 considered statistically significant.

### 2.6 Ethical considerations

This study was approved by the Ethics Committee of Ningxia Medical University (Approval No. NXMU Ethics 2023-044). Before recruiting participants, the research team communicated with the relevant clinical departments to obtain formal authorization for conducting the survey. Researchers provided participants with a comprehensive explanation of study objectives, practical value, and implementation procedures. Personal and clinical data were kept confidential to protect participants' privacy, ensuring the non-disclosure of personally identifiable information at any stage of the study. All participants' involvement was entirely voluntary. Each participant was fully informed about the study and signed an informed consent form prior to data collection.

## 3 Results

### 3.1 Participant characteristics and univariate analysis of fear of disease progression

Among the 300 stroke patients included, 228 were men (76.0%); 148 were aged ≥60 years (49.3%); 159 were residents of urban areas (53.0%); 112 had primary school and below education (37.3%); 256 were married (85.3%); 140 were employed (46.7%); 160 had household incomes less than their expenses (53.3%); 171 paid for medical expenses out of pocket (57.0%); 180 were mildly dependent in activities of daily living (60.0%); 271 had ischemic stroke (90.3%); and 152 reported being unfamiliar with stroke-related information (50.7%). The mean FoP score was 30.34 ± 8.56. Other general characteristics are shown in Table 1. In univariate analyses, age, place of residence, employment status, household economic status, medical payment method, ADL, stroke type, and stroke-related knowledge were all significantly associated with FoP (all *P* < 0.05).

**Table 1:** Univariate analysis of FoP in stroke patients.

Variable	Category	*n* (%)	FoP scroes($\bar{x}$ ±s)	t/F	*P*
Gender	Male	228 (76.0)	29.90 ± 7.97	1.395	0.166
	Female	72 (24.0)	31.72 ± 10.14		
Age (years)	18-44	36 (12.0)	32.56 ± 7.97	9.474	< 0.001
	45-59	116 (38.7)	32.35 ± 8.47		
	≥60	148 (49.3)	28.22 ± 8.30		
Place of residence	Urban	159 (53.0)	28.94 ± 8.20	3.034	0.003
	Rural	141 (47.0)	31.91 ± 8.71		
Education level	Primary school and below	112 (37.3)	30.14 ± 8.59	0.082	0.970
	Junior high school	95 (31.7)	30.23 ± 8.69		
	Technical secondary/high school	58 (19.3)	30.79 ± 8.04		
	University and above	35 (11.7)	30.49 ± 9.30		
Marital status	Unmarried	9 (3.0)	29.78 ± 6.10	0.909	0.404
	Married	256 (85.3)	30.60 ± 8.68		
	Divorced/Widowed	35 (11.7)	28.54 ± 8.18		
Employment status	Employed	140 (46.7)	33.34 ± 9.02	18.435	< 0.001
	Retired	78 (26.0)	27.04 ± 6.91		
	Unemployed	82 (27.3)	28.35 ± 7.46		
Household economic status	Income>Expenditure	36 (12.0)	27.47 ± 7.22	10.494	< 0.001
	Break-even	104 (34.7)	28.18 ± 8.21		
	Income < Expenditure	160 (53.3)	32.38 ± 8.58		
Medical payment method	Insured	129 (43.0)	29.12 ± 8.49	2.143	0.033
	Uninsured	171 (57.0)	31.25 ± 8.53		
ADL	Independent	22 (7.3)	28.05 ± 8.37	7.650	< 0.001
	Mildly dependent	180 (60.0)	29.08 ± 8.13		
	Moderately dependent	69 (23.0)	31.84 ± 8.94		
	Severely dependent	29 (9.7)	36.28 ± 7.57		
Stroke types	Hemorrhagic	29 (9.7)	35.38 ± 8.06	3.395	< 0.001
	Ischemic	271 (90.3)	29.80 ± 8.45		
Knowledge of stroke-related information	Very familiar	3 (1.0)	29.67 ± 8.62	6.530	< 0.001
	Moderately familiar	52 (17.3)	27.73 ± 8.07		
	Slightly familiar	93 (31.0)	28.41 ± 8.11		
	Unfamiliar	152 (50.7)	32.42 ± 8.55		

### 3.2 Hierarchical linear regression analysis of perceived social support and its association with fear of disease progression in stroke patients

The assumptions of linear regression were checked prior to the analysis. Residuals were visually inspected using Q-Q plots and scatterplots, which indicated acceptable normality and homoscedasticity. A hierarchical linear regression analysis was performed with FoP score as the dependent variable. PSS was added as the primary explanatory variable, and covariates were selected based on statistical significance in univariate analyses. Model 1 included age, residence, employment status, household economic status, medical payment method, ADL, stroke type, and stroke-related knowledge as covariates, with an adjusted *R*^2^ of 0.215 (*F* = 6.465, *P* < 0.001). Retired status, unemployed status, and severe ADL dependence were associated with FoP in Model 1, whereas the other covariates were not. In Model 2, PSS was additionally included, yielding an adjusted *R*^2^ of 0.309. The addition of PSS explained an additional 9.1% of the variance in FoP (Δ*R*^2^ = 0.091, *F*-change = 39.544, *P* < 0.001). In Model 2, higher PSS remained significantly associated with lower FoP scores (*B* = −0.223, *P* < 0.001). Compared with employed patients, unemployed patients had significantly lower FoP scores (*B* = −3.188, *P* = 0.008), while the difference between retired and employed patients was no longer statistically significant (*P* = 0.099). Severe ADL dependence was associated with higher FoP scores compared with independence (*B* = 4.921, *P* = 0.035; Table 2). In addition, in the sensitivity analysis including all variables from the univariate analyses, the association between PSS and FoP remained consistent (*B* = −0.214, *P* < 0.001, adjusted *R*^2^ = 0.323), supporting the robustness of the findings.

**Table 2:** Hierarchical linear regression analysis of factors associated with FoP.

Model	Variables	Category	B	S.E.	β	*t*	*P*
Model 1	(Constant)		27.447	6.448		4.257	< 0.001
	Age	18-44					
		45-59	−0.337	1.484	−0.019	−0.227	0.820
		≥60	−2.315	1.674	−0.135	−1.383	0.168
	Place of residence	Urban					
		Rural	0.838	1.005	0.049	0.834	0.405
	Employment status	Employed					
		Retired	−3.011	1.485	−0.155	−2.028	0.043
		Unemployed	−4.746	1.237	−0.247	−3.837	< 0.001
	Household economic status	Income>Expenditure					
		Break-even	−0.148	1.553	−0.008	−0.095	0.924
		Income < Expenditure	1.447	1.665	0.084	0.870	0.385
	Medical payment method	Insured					
		Uninsured	1.327	0.948	0.077	1.400	0.163
	ADL	Independent					
		Mildly dependent	0.889	1.766	0.051	0.503	0.615
		Moderately dependent	3.294	1.955	0.162	1.685	0.093
		Severely dependent	7.691	2.437	0.266	3.156	0.002
	Stroke types	Hemorrhagic					
		Ischemic	0.134	1.831	0.005	0.073	0.942
	Knowledge of stroke-related information	Very familiar					
		Moderately familiar	−1.204	4.558	−0.053	−0.264	0.792
		Slightly familiar	−2.135	4.514	−0.116	−0.473	0.637
		Unfamiliar	1.556	4.530	0.091	0.344	0.731
Model 2	(Constant)		39.455	6.345		6.219	< 0.001
	Age	18-44					
		45-59	−0.021	1.394	−0.001	−0.015	0.988
		≥60	−1.410	1.577	−0.082	−0.894	0.372
	Place of residence	Urban					
		Rural	0.915	0.943	0.053	0.971	0.333
	Employment status	Employed					
		Retired	−2.314	1.397	−0.119	−1.656	0.099
		Unemployed	−3.188	1.187	−0.166	−2.687	0.008
	Household economic status	Income>Expenditure					
		Break-even	−0.218	1.457	−0.012	−0.149	0.881
		Income < Expenditure	0.717	1.566	0.042	0.458	0.647
	Medical payment method	Insured					
		Uninsured	1.192	0.890	0.069	1.339	0.182
	ADL	Independent					
		Mildly dependent	0.357	1.659	0.020	0.215	0.830
		Moderately dependent	2.041	1.845	0.100	1.106	0.270
		Severely dependent	4.921	2.329	0.170	2.113	0.035
	Stroke types	Hemorrhagic					
		Ischemic	0.145	1.718	0.005	0.085	0.933
	Knowledge of stroke-related information	Very familiar					
		Moderately familiar	0.439	4.285	0.019	0.103	0.918
		Slightly familiar	−1.603	4.237	−0.087	−0.378	0.706
		Unfamiliar	1.145	4.251	0.067	0.269	0.788
PSS		−0.223	0.035	−0.349	−6.288	< 0.001

Model fit statistics:

Model 1: *R*^2^ = 0.255, adjusted *R*^2^ = 0.215, *F* = 6.465, *P* < 0.001;

Model 2: *R*^2^ = 0.346, adjusted *R*^2^ = 0.309, Δ*R*^2^ = 0.091, *F*-change = 39.544, *P* < 0.001.

### 3.3 Restricted cubic spline and exploratory threshold effect analysis of perceived social support and fear of disease progression

The RCS model was adjusted for covariates that were statistically significant in the multivariate regression models, including employment status and ADL, with 4 knots placed at the 5th, 35th, 65th, and 95th percentiles of the PSS distribution, selected based on the minimum AIC. RCS analysis suggested a nonlinear association between PSS and FoP (*P*_foroverall_ < 0.001; *P*_fornonlinearity_ = 0.023; Figure 1). The exploratory two-piecewise model identified a change point at a PSS score of 67 and fit the data better than the single-line model (likelihood-ratio *P* = 0.011). At PSS < 67, higher PSS was associated with lower FoP, corresponding to an average decrease of 0.319 points in FoP for each one-point increase in PSS (slope = −0.319, 95% CI −0.396 to −0.241; *P* < 0.001). At PSS ≥ 67, the slope estimate was 0.304 (95% CI −0.142 to 0.751; *P* = 0.182; Table 3). In this study, 67 patients (22.3%) had PSS scores of 67 or above.

**Figure 1:**
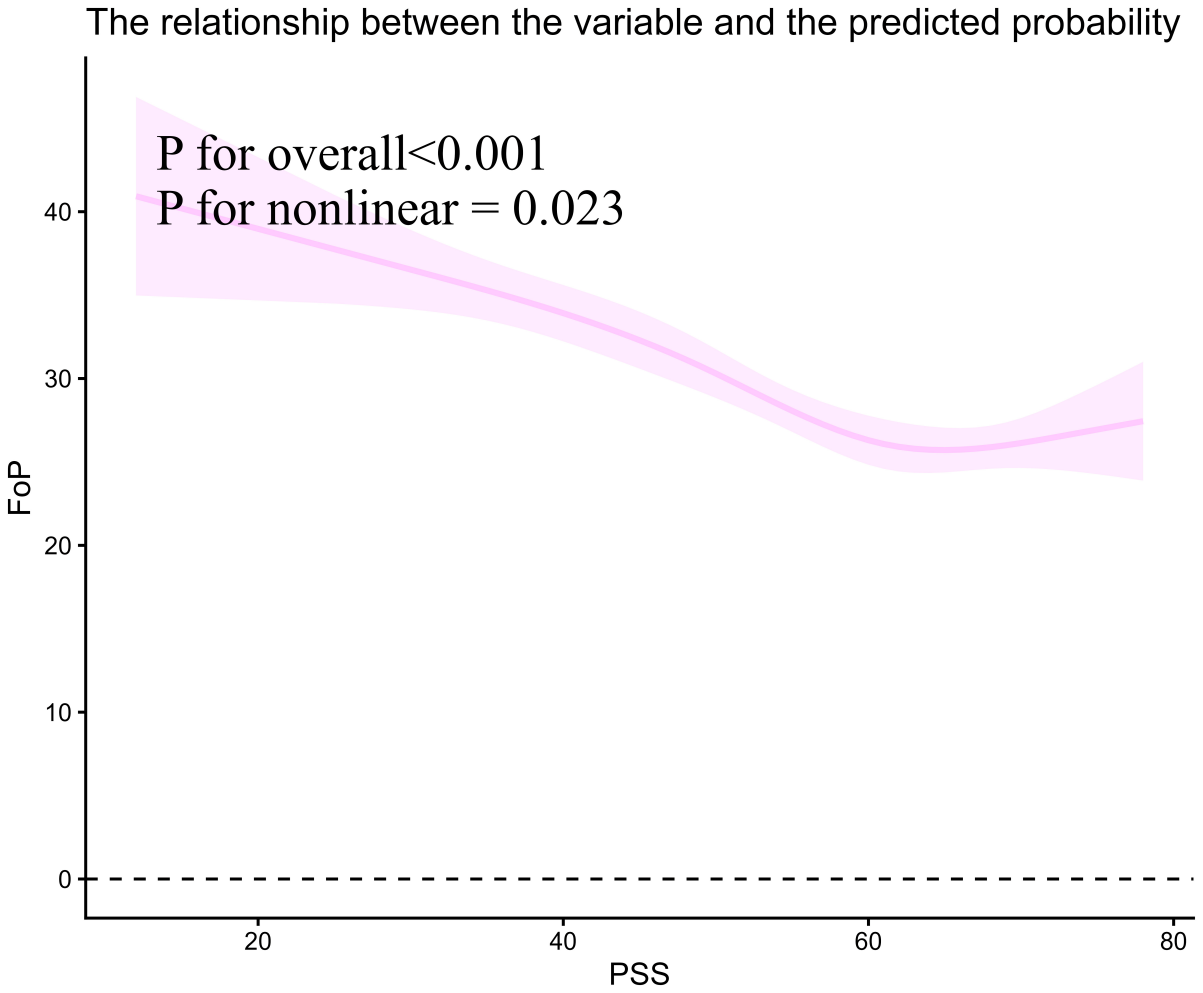
Restricted cubic spline analysis of the association between PSS and FoP.

**Table 3:** Exploratory threshold effect analysis of the association between PSS and FoP.

Model		*B* (95%CI)	*P*
Model 1	Standard linear effect	−0.264 (−0.33, −0.198)	< 0.001
Model 2	Change point of PSS	67	
	PSS < 67	−0.319 (−0.396, −0.241)	< 0.001
	PSS≥67	0.304 (−0.142, 0.751)	0.182
	Likelihood ratio test	-	0.011

## 4 Discussion

### 4.1 Factors associated with fear of disease progression in stroke patients

The mean FoP score in the present study was 30.34 ± 8.56, which is broadly consistent with the mean FoP score of 29.11 ± 6.72 reported by Liu et al. (29) in a cross-sectional study of 235 Chinese stroke patients. Moreover, a recent systematic review and meta-analysis including 20 studies and 7,156 patients also indicated that FoP is a common psychological concern among Chinese stroke survivors (41). However, the FoP score in the present study was lower than that reported by Yi et al. (42). This discrepancy may be partly attributable to the higher proportion of older patients in our sample. Compared with younger and middle-aged patients, older patients often have richer life experience and are mostly retired or receiving home care; these factors may be associated with greater psychological resilience to illness and lower fear of disease progression (41). In contrast, younger and middle-aged individuals are often in a critical period of family and career development, and a stroke event during this stage may substantially disrupt their work, family, and social lives, potentially increasing psychological distress (43). Moreover, the sudden onset of stroke, combined with the need for long-term rehabilitation and treatment, may impose a dual burden that contributes to a higher level of fear of disease progression in this population (42).

In the univariate analyses, age, place of residence, family economic status, medical payment method, ADL, stroke type, and stroke-related knowledge were associated with FoP. However, after adjustment for other covariates, only employment status (unemployment), ADL (severe dependence), and PSS remained independently associated with FoP. Evidence regarding employment and FoP after stroke is not entirely consistent. Liu et al., for example, found unemployment to be associated with higher FoP (29), whereas a recent prospective study of first-ever stroke also identified employment status as one of the factors associated with different FoP trajectories (44). In the present study, employed patients had higher FoP scores than unemployed and retired patients. Some indirect evidence from cancer populations has reported similar employment-related concerns (45). Although the study populations differ slightly between the present study and this previous study, the core contributors to fear of disease progression—including symptom-related distress, financial burden (46), and significant changes in life roles—are common across various patient populations and may be broadly applicable. Additionally, work commitments may interfere with treatment compliance and may be associated with worries about disease outcomes. This finding is consistent with studies by Janz et al. (47) and Shay et al. (48). Importantly, return to work after stroke is a complex rehabilitation outcome. Systematic reviews have shown that functional independence, stroke severity, communication impairment, and occupational characteristics are important prognostic factors for return to work (49), while qualitative evidence indicates that working-age stroke survivors frequently experience substantial challenges related to employment and work reintegration (50). Thus, one possible explanation for the present finding is that patients who remain employed may simultaneously face concerns regarding functional recovery, work continuity, and occupational responsibilities. These compounded concerns may contribute to higher FoP among employed patients. Therefore, healthcare professionals should pay particular attention to the dual concerns of employed stroke patients regarding rehabilitation prognosis and return to work, and should identify their psychological distress at an early stage. In addition, individualized vocational rehabilitation guidance could be provided based on patients' functional recovery, with staged rehabilitation-to-employment recommendations for those who wish to resume work.

ADL was another factor independently associated with FoP. This finding is consistent with recent evidence showing that lower functional independence, assessed using the Barthel Index, was associated with a higher FoP profile among ischemic stroke survivors (13). Reduced functional independence may reflect greater disease severity and poorer prognosis. Patients may therefore become more concerned about the severe consequences of stroke progression or recurrence—such as paralysis, aphasia, and dementia—as well as increased care dependency, which may heighten their psychological burden. This burden may, in turn, weaken patients‘ confidence in treatment and rehabilitation and impose substantial social and psychological strain, which may be associated with greater fear related to disease progression (41). Accordingly, clinically, joint assessment of functional status and psychological concerns may help identify patients who need more comprehensive rehabilitation and psychosocial support, which could inform the development of appropriate interventions based on patients' functional status and psychological condition, with the aim of enhancing psychological resilience, reducing psychological burden, and lowering FoP levels.

### 4.2 Association between perceived social support and fear of disease progression in stroke patients

PSS was also independently associated with lower FoP. This finding is supported by an increasing body of stroke-specific evidence. A recent systematic review and meta-analysis reported that greater social support was associated with lower FoP among Chinese stroke patients (41). Similarly, Yang et al. (51) provided further evidence for a meaningful association between PSS and FoP or FoP-related psychological processes. From a theoretical perspective, the association may be compatible with the stress-buffering model, which proposes that perceived availability of interpersonal resources can mitigate psychological responses to stressful circumstances (52). In stroke patients, high levels of social support may provide emotional reassurance, social connection, and practical assistance, such as company, help with meals, or financial support (53). These resources may reduce distress and uncertainty during treatment and rehabilitation (28). Second, patients who perceive greater support may have better access to health information through their social networks, which may facilitate illness understanding and adherence to recommended health behaviors (54). Therefore, healthcare providers may consider assessing patients' psychosocial needs, provide tailored information and psychological support, and encourage family members to actively participate in disease management to strengthen family support, thereby facilitating better treatment adherence and recovery. In addition, community-based rehabilitation services could be used to build peer support platforms and integrate multiple resources, helping patients establish and maintain a stable social support network.

An important finding was the nonlinear pattern of the association between PSS and FoP. In the exploratory piecewise analysis, higher PSS remained associated with lower FoP below a exploratory change point of approximately 67. Namely, each one-point increase in PSS was associated with a 0.319-point decrease in FoP (95% CI: −0.396 to −0.241, *P* < 0.001). In contrast, the slope above 67 was not statistically significant and was estimated with substantially less precision. One possible explanation is that in this study, only 67 participants (22.3%) had PSS scores of 67 or higher, which reduced the information available to characterize the association in this range and contributed to the wide confidence interval. The apparent change in slope may reflect a true nonlinear relationship, sampling variability, or both. Therefore, larger samples with more participants at higher PSS levels, together with internal validation and external replication, are needed before the estimated value of 67 can be considered stable or clinically meaningful. Therefore, for stroke patients with insufficient PSS, nurses and multidisciplinary teams could prioritize assessing family involvement, peer support, patient-provider communication, access to rehabilitation information, and community support resources. They could also enhance patients' perception of available support through family-participatory care, peer support, continuity of care, and individualized disease education. For patients with PSS ≥ 67, future studies with larger samples of patients with high PSS levels are needed to clarify the pattern of the association and to determine whether a clinically meaningful threshold exists.

### 4.3 Limitations

Several limitations of this study should be acknowledged. First, the cross-sectional design precludes inference of causality or examination of temporal changes in the relationships between variables. Second, convenience sampling was used to recruit patients from two tertiary hospitals in Yinchuan, which may introduce selection bias and limit the generalizability of the findings to stroke patients in other regions or different healthcare settings. Future studies with multicenter sampling and broader patient populations are needed to improve the representativeness of the sample. Third, stroke-related knowledge was assessed using a single study-specific self-reported item rather than a validated knowledge scale. This may have affected the accuracy of the knowledge assessment. Future studies could use validated instruments to assess stroke-related knowledge more accurately and comprehensively. Finally, only 67 patients (22.3%) had PSS scores of 67 or above. This relatively small subgroup likely contributed to the wide confidence intervals and unstable estimates observed in this segment of the RCS curve. Larger multicenter, large-sample prospective studies are needed to evaluate the reproducibility and clinical relevance of the observed nonlinear pattern and exploratory change point in the future.

## 5 Conclusions

This study found evidence of a nonlinear association between PSS and FoP among stroke patients, with a exploratory change point at a PSS score of 67. Higher PSS was associated with lower FoP below this point, whereas the association above 67 was not statistically significant and was imprecise, requiring validation in larger multicenter and prospective studies. In addition, employment status (unemployed) and ADL (severely dependent) were also independently associated with FoP. These findings may help guide more comprehensive psychosocial assessment for stroke patients at risk of FoP.

## Data Availability

The raw data supporting the conclusions of this study are not publicly available due to privacy and ethical restrictions. The data are available from the corresponding author upon reasonable request.
